# Development of an Optimized Medium, Strain and High-Throughput Culturing Methods for *Methylobacterium extorquens*

**DOI:** 10.1371/journal.pone.0062957

**Published:** 2013-04-30

**Authors:** Nigel F. Delaney, Maria E. Kaczmarek, Lewis M. Ward, Paige K. Swanson, Ming-Chun Lee, Christopher J. Marx

**Affiliations:** 1 Department of Organismic and Evolutionary Biology, Harvard University, Cambridge, Massachusetts, United States of America; 2 Faculty of Arts and Sciences Center for Systems Biology, Harvard University, Cambridge, Massachusetts, United States of America; Iowa State University, United States of America

## Abstract

*Methylobacterium extorquens* strains are the best-studied methylotrophic model system, and their metabolism of single carbon compounds has been studied for over 50 years. Here we develop a new system for high-throughput batch culture of *M. extorquens* in microtiter plates by jointly optimizing the properties of the organism, the growth media and the culturing system. After removing cellulose synthase genes in *M. extorquens* strains AM1 and PA1 to prevent biofilm formation, we found that currently available lab automation equipment, integrated and managed by open source software, makes possible reliable estimates of the exponential growth rate. Using this system, we developed an optimized growth medium for *M. extorquens* using response surface methodologies. We found that media that used EDTA as a metal chelator inhibited growth and led to inconsistent culture conditions. In contrast, the new medium we developed with a PIPES buffer and metals chelated by citrate allowed for fast and more consistent growth rates. This new *Methylobacterium* PIPES (‘MP’) medium was also robust to large deviations in its component ingredients which avoided batch effects from experiments that used media prepared at different times. MP medium allows for faster and more consistent growth than other media used for *M. extorquens*.

## Introduction

The α-proteobacterium *Methylobacterium extorquens* has served for over 50 years [1] as the premier model system for uncovering the genetic basis of growth on C_1_ compounds (i.e., methylotrophy) [2]. A significant number of genetic tools have been developed for *M. extorquens* over the past decade [3], [4], [5], [6], [7], and complete genome sequences are now available for four strains of *M. extorquens*, as well as four genomes of other species in the genus [8], [9]. This combination of genetic tools and genomic information has catalyzed experimental and computational analysis of systems-level properties [10], [11], as well as the expansion of research into topics ranging from the natural ecology of *Methylobacterium* as a leaf epiphyte [12] to the adaptation of *M. extorquens* strains in the laboratory [13], [14], [15].

To facilitate quantitative analysis of the physiology of *M. extorquens,* we sought to develop a system for high-throughput growth rate assays. Studies using high-throughput methods to analyze growth rates have typically grown facultatively anaerobic organisms such as *Saccharomyces cerevisiae* or *Escherichia coli* in microtiter plate cell culture systems (see for example [16], [17]). However, as *M. extorquens* are strict aerobes, this makes growth in traditional microtiter plate systems challenging, because oxygen transfer can be poor and stratification can readily occur [18], [19]. An earlier attempt to grow *M. extorquens* AM1 strains in 96-well plates [15] resulted in informative qualitative patterns between strains, but the growth rate was extremely inconsistent through time and slower than what had been measured in flasks [14]. We therefore asked whether a combination of currently available products could be used or modified to allow for high throughput measurement of cultures of *Methylobacterium*.

Broadly speaking, automatic growth curve systems can be divided into two groups [19]. The first type of system is completely integrated, whereby an incubator, shaker and plate reader are all contained in one physical instrument. Although such systems are typically easy to employ because they do not require integration of different instruments, they usually have limited capacity and can read and incubate only 1–2 plates at a time, and thus ∼100–200 cultures simultaneously. The second type of systems are those which have a physically separate shaking incubator where many plates can be grown, and a robotic arm which periodically moves plates from the incubator to a plate reader for measurements. A system like this was used in the robotic scientist project used to study *S. cerevisiae* [20], and has also been successfully employed in the measurement of growth rates of *E. coli* [17]. Because these systems allow for the measurement of several plates simultaneously, we used a collection of instruments with a separate incubator, plate reader and robotic arm as the starting point for this research.

High throughput growth rate assays not only require a robotic system that can take the necessary measurements, but also require culture conditions and a medium that allows for robust, repeatable growth rate measurements. A well-designed medium should not introduce biases or batch effects as it is remade or different reagent stocks are used. The medium formulation should also maintain a reasonably consistent environment throughout the growth cycle, and appropriately buffer any pH changes due to excretion or consumption during culture growth. At the outset of this study, it quickly became clear that the medium our lab had historically used, which is a variant of ‘Hypho’ medium (Text S1), did not fit these criteria. This medium uses phosphate as the pH buffer and contains an EDTA-chelated trace metal mix [13].

We experienced multiple problems with this medium, some of which were due to metal limitation [21]. In some cases, freshly prepared medium did not support growth until the medium had aged for several days and as a result different batches of media would produce distinctly different growth rates. We also found that measured growth rates differed depending on if the culture was being grown in plastic microtiter plates or in glassware. Further, during a long-term evolution experiment using this old medium recipe, cultures evolved to overcome trace metal limitations. One beneficial mutation that appeared in an experiment increased expression of a metal transporter [21]. This mutation specifically increased cobalt uptake, which is necessary for synthesis of the vitamin B_12_ that *M. extorquens* AM1 uses in a glyoxylate-regeneration pathway [21]. An independent analysis based upon quantitative metabolomics came to the same conclusion that this medium is limiting for cobalt during methylotrophic growth [22].

As cobalt and other trace metals are present at quantities typical of other media, we suspected the problems we observed were largely caused by the use of EDTA in the trace metal solution (Text S1), where it functions to chelate metal cations in the media. EDTA has long been known to inhibit the growth of *M. extorquens* on methanol [23], [24], [25], and may sequester the metal cations so they are inaccessible to the cells. Consistent with this hypothesis, treating the media with light to degrade some of the EDTA allowed for improved growth [21]. However, we had not previously noticed these effects because robust growth could often be observed after the medium had aged by just a few days, the cells were less sensitive to the age of the medium when grown in glass, and quantitative differences were hard to detect without a robotic system. Collectively, it became clear that our traditionally used Hypho medium could not robustly support growth.

We therefore sought to create a new medium, ensure that no components in it were limiting or inhibiting growth, and show that it was robust to deviations in the component concentrations. There are two common methodological approaches taken to optimize a medium formulation for these criteria. The first is the one-factor-at-a-time method, which determines how a change in each component of the medium affects the growth dynamics by separately varying each component while holding the levels of the other components constant. For example, Neidhardt used this approach to develop a medium for enterobacteria by independently varying the levels of various nutrients and salts to determine their effects [26]. One advantage of this approach is that it is simple to implement and interpret. However, it can fail to discover interactions, or effects that are caused by simultaneous changes to multiple components. In contrast, response surface methodologies [27] take a multi-factorial approach to designing an optimal medium. Components of the medium are simultaneously varied in one large experiment rather than multiple separate experiments and statistical models are subsequently fit to estimate the effect of a varying a component either independently or in concert with other components. This approach is thus able to capture interactions between variables and use quantitative models to guide the optimization [27], [28].

While designing a new media, we used this response-surface approach and in addition to varying the concentrations of the components in the original media, we also wanted to evaluate the use of alternative pH buffers as well as the effect of the total buffer concentration. Phosphate pH buffers are commonly used in media. They are easy to prepare and allow for the pH of a medium to be simply changed by varying the proportion of the dibasic and monobasic phosphate salts [29]. However, phosphate buffers have previously been found to inhibit the growth of some microorganisms [30], including *Methylobacterium* [31]. The effects of an alternative buffer such as PIPES, which performs well for Enteric bacteria [26], were unknown. Additionally, although past medium formulations for *Methylobacterium* have either used the strong chelator EDTA [14] or no strong chelator [32] (though phosphate buffers can act as a weaker chelator), we wanted to evaluate the effect of the alternative chelators NTA and citrate.

Here we report the results of these studies and a new *Methylobacterium* PIPES (‘MP’) medium that contains a citrate-chelated, trace metal solution with seven metals (‘C7’). Furthermore, we present the creation of genetically modified *M. extorquens* strains that do not clump while growing and thus allow for substantially more consistent measurements of a culture’s growth rate. With this combination of new medium and strains, cultures growing in our 48-well microtiter plate culture system can reliably begin and maintain a phase of exponential growth, allowing for growth rates to be measured accurately across hundreds, or even thousands, of cultures simultaneously. The new MP medium outperforms previous formulations and provides stable, rapid growth that will greatly promote the increasingly quantitative and systems-wide physiological analyses of *M. extorquens*.

## Methods and Results

### General Materials and Methods

#### Strains

The two strains used in this study were *M. extorquens* PA1 [8], [33] (henceforth ‘PA1’), which grows well on succinate but cannot double on methylamine in less than 15 hours, and *M. extorquens* AM1 [8], [34] (henceforth ‘AM1’), which grows quickly on both succinate and methylamine. When the experiments tested AM1 on both succinate and methylamine and PA1 on succinate, we simply write that “all strains were tested on all substrates”.

#### Growth rate measurements

Growth rate measurements were performed using a robotic system composed of a shaking incubator that holds multi-well plates (Liconic USA LTX44 with custom fabricated cassettes) and a series of robotic instruments that can move these plates at regular intervals to a Perkin-Elmer Victor2 plate reader for optical density (OD_600_, simply ‘OD’ hereafter) readings. A video showing the system and how the plates are moved is available at http://www.evolvedmicrobe.com/LabAutomation.html. The instruments were integrated and managed by Clarity, a recently described open source software program [35]. The entire robotic system is contained in a temperature- and humidity-controlled room set to 30°C and 80% relative humidity. The OD readings output by these instruments were parsed and fitted to an exponential model of cell growth using a custom data analysis software package designed for this purpose, Curve Fitter (http://www.evolvedmicrobe.com/CurveFitter/).

#### Construction of Δ*cel* strains

Allelic exchange plasmids for generating strains were constructed from pCM433, a *sacB-*based suicide plasmid, as described previously [36], [37]. PCR products of sequences upstream and downstream of a region containing genes suggested to be involved in cellulose synthesis were sequentially cloned in to pCM433 to create separate cloning vectors for PA1 and AM1. The genomic region flanked by the amplified regions contains genes that are numerically annotated in the reference AM1 genome [4] as META1_1167, META1_1168, META1_1169 and META1_1170). The primer pair Forward: GGACGTCGCTCCTACTCGACCAAGTTCAACG/Reverse: GCATATGAGATCTCGATTTCCGGATGCGTTCACGAC was used to amplify the upstream region and the pair Forward: CCATATGCACGAAGTGAGGACGCCTTCGATCTCTAC/Reverse: GGAGCTCTTCGAGCACGTCGATGAACAGCA was used to amplify the downstream region from both AM1 and PA1. The upstream PCR products from AM1 and PA1 were ligated into pCM433 following digestion by *Nde*I/*Aat*II. This created additional plasmids that following a subsequent digestion by *Sac*I/*Nde*I the downstream region was ligated into. This created the allelic exchange plasmid pLW17 for generating an AM1 strain (Genbank: KC820640) and plasmid pLW18 for generating a PA1 strain (Genbank: KC820641). These two plasmids were then respectively used to obtain unmarked Δ*cel* strains CM2720 and CM2730. The deletion in both strains was confirmed by sequencing.

#### Growth medium

Hypho medium was the starting point for the optimization experiments and for reference its recipe is given in text S1. The C7 metal mix we use in these experiments along with the complete MP medium recipe that came from this work is given in table S1. When an experiment varied the levels of the C7 solution, we describe any alternate concentration as a multiple of the concentration in the final recipe. All media were tested at a pH of 6.75. The substrate concentrations varied slightly over the course of this research as we made small adjustments (15 or 17 mM methylamine, and either 5 or 5.6 mM succinate was used). We selected concentrations that allowed the cells to grow up to an equivalent maximum OD on both substrates, and that were as high as possible while avoiding a noticeable decline in the growth rate. When statistical models are employed, they are described using the standard formulas for the R/S languages (described online or at pg. 329 in [2]).

The optimization of the medium recipe was performed by a series of experiments whose central question and simplified conclusions are outlined in Table 1. The specific methods and analysis of each experiment is described in the following subsections.

**Table 1:** Summary of experimental questions and findings.

Experiment Number	Question	Answer
1	Do 96 well plates allow for effective growth of *M. extorquens*?	Unlike 48 well plates they do not provide adequate shaking.
2	Can clumping be eliminated?	Deleting the *cel* operon created strains that do not form clumps in batch culture.
3	Which chelators work best for *M. extorquens* media?	EDTA and NTA do not work well, but using citrate or no chelator does.
4	Are phosphate salts or PIPES a better pH buffer?	At higher buffer concentrations (48 mM) the phosphate salts are distinctly worse. At lower concentrations (30 mM) PIPES is still slightly better.
5	Does the sterilization method used for the C7 metal solutionhave an effect on the measured growth rates?	No, equivalent growth is seen when the solution is autoclaved, filtered or used without sterilization.
6	Can *M. extorquens* use citrate?	No, the citrate chelator does not support growth.
7	Can changing the concentrations of any of the medium components lead to a better medium?	No, the MP recipe is optimal.
8	How does the new MP media compare to some othermedia used for *M. extorquens*?	MP medium permitted faster, more stable growth than previously used media.

#### 1. Initial tests and use of 48-well plates

We first tested whether we could reliably measure *M. extorquens* exponential growth rates using a robotic measurement system under similar conditions to those used by previous investigators with *E. coli* [3]. AM1 cultures were grown in a Microtest 96-well tissue culture treated plates (Falcon-35-3072) using buffered medium comprised of 14.5 mM of K_2_HPO_4_, 18.8 mM of NaH_2_PO_4_, 8 mM ammonium sulfate, 20 µM calcium chloride and the C7 metal mix that was left unchelated (i.e., no citrate) with 17 mM methylamine·HCL added to the base medium. The mixture was aliquoted in 160 µL portions into wells of a 96-well plate. The growth curves from the initial tests in 96 well plates showed huge deviations from the exponential model and were exceptionally noisy. We concluded that 96-well plates were inadequate for sustained exponential growth of *M. extorquens*. We re-evaluated this conclusion at the end of this project, after optimizing our strains and media, by again growing *M. extorquens* in MP media and in 96-well plates (Fig S1). Although exponential growth could then be achieved, relative to a 48-well plate grown simultaneously with the same inoculum, the average estimated growth rate was 7% slower and the std. error was 50% larger.

**Figure 1:**
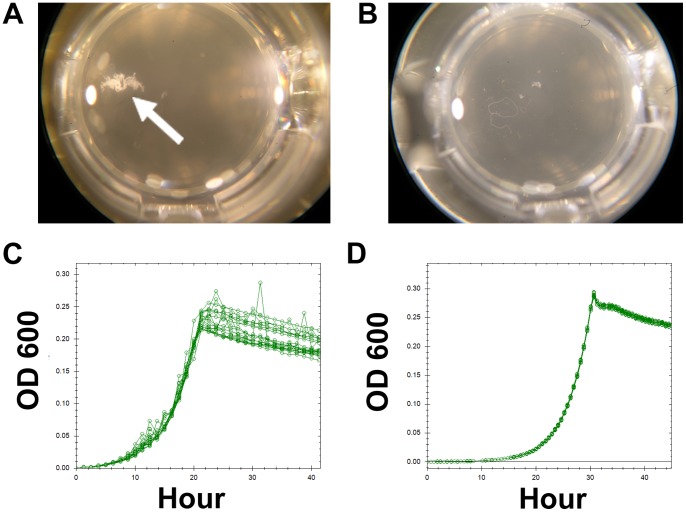
A comparison of the growth characteristics of *M.extorquens* with and without the cellulose genes removed. The top row shows pictures of individual wells after growth with (A) or without (B) the cellulose genes. A large clump of the wild-type strain is indicated by the white arrow. Such clumps were not found for AM1Δ*cel*, but occasionally small strands such as those shown in B can appear. The bottom row (C and D) shows example growth curves obtained for 12 replicates with or without the cellulose locus. The OD readings of wild-type were noisy and irregular (C), whereas the AM1Δ*cel* growth dynamics were more consistent (D).

In contrast, we found that 48-well plates did allow for adequate mixing and consistent exponential growth (Fig 1D). We therefore altered the robotic system by installing new custom-built racks so that it could use 48-well plates (CoStar-3548) instead of 96-well plates. In contrast to the 96-well plates where the medium did not appear to move within the well, the media in the 48-well plates rhythmically swirled around. We also tested a second type of 48-well plate, from the Cellstar line made by Greiner Bio-One (Catalog #677 102). Surprisingly, although medium in the CoStar plates visibly swirled while shaking, the meniscus in the Cellstar plates, as in the 96-well plates, stayed at approximately the same level and did not appear to move; correspondingly, cultures grew much more poorly in Cellstar plates. For all future work in this paper, we grew the cultures in CoStar plates in 640 µL per well with the incubator shaking at 650 RPM, as growth and the swirling of the liquid appeared to be as good or better than the range of other values tested.

#### 2. Creation of a cellulose deletion strain

We noticed that cultures growing in microtiter plates formed clumps that moved around inside the wells (Fig 1A) and contributed to noise in the OD readings. Other researchers had found that an AM1 strain with a transposon insertion into a cellulose synthase gene (*celB*) did not clump as frequently or as severely as the wild-type (A. Stöver and M. Lidstrom, personal communication). To replicate this effect in our strains without introducing the markers associated with the transposon insertion, we removed a 7,183 bp region containing this gene and two related genes. These three genes are suggested to be involved in cellulose synthesis–*celA*, *celB* and *celC*– as well as a portion of a gene with an unknown function. For simplicity, hereafter we refer to the strains created as AM1Δ*cel* and PA1Δ*cel* (CM2720 and CM2730, respectively).

Growth dynamics on the robotic system were strikingly different between the Δ*cel* strains compared to the corresponding Cel^+^ wild-type cultures (Figs. 1C, 1D). The Δ*cel* strains showed significantly more stable curves without the sharp spikes in OD measurements previously observed (Fig 1D), and thus from this point forward all optimization was performed with these two strains.

#### 3. Testing different metal chelators

We previously found that when EDTA was used to chelate metals in our medium with either methanol or methylamine used as the substrate, cultures often grew slowly [21], and sometimes would not grow at all in 48-well plates. To test other chelators and quantify how they affected the growth of *M. extorquens,* we compared the growth rates of both AM1 and PA1 Δ*cel* strains on methylamine, succinate and methanol, using five different chelator treatments. We tested three chelators (EDTA, NTA, and citrate), as well as two unchelated metal mixes that were prepared either immediately before testing or several months prior to the experiment. Trace metal solutions with different chelator treatments were prepared by adding each chelator to a solution otherwise identical to the C7-metal mix solution (Table S1) except made by excluding citrate from the recipe so that the base solution did not already contain a chelator. Five concentrations of the chelator concentration (given as a percentage of total moles of chelator relative to total moles of metal ions in the solution) were tested for both EDTA and NTA: 5%, 25%, 50%, 100% and 200%. Citrate was restricted to three levels: 50%, 100% and 150%. Excepting the trace metal treatments, all media tested were identical to a Hypho medium recipe made while excluding that medium’s own trace metal solution (Text S1).

**Figure 2:**
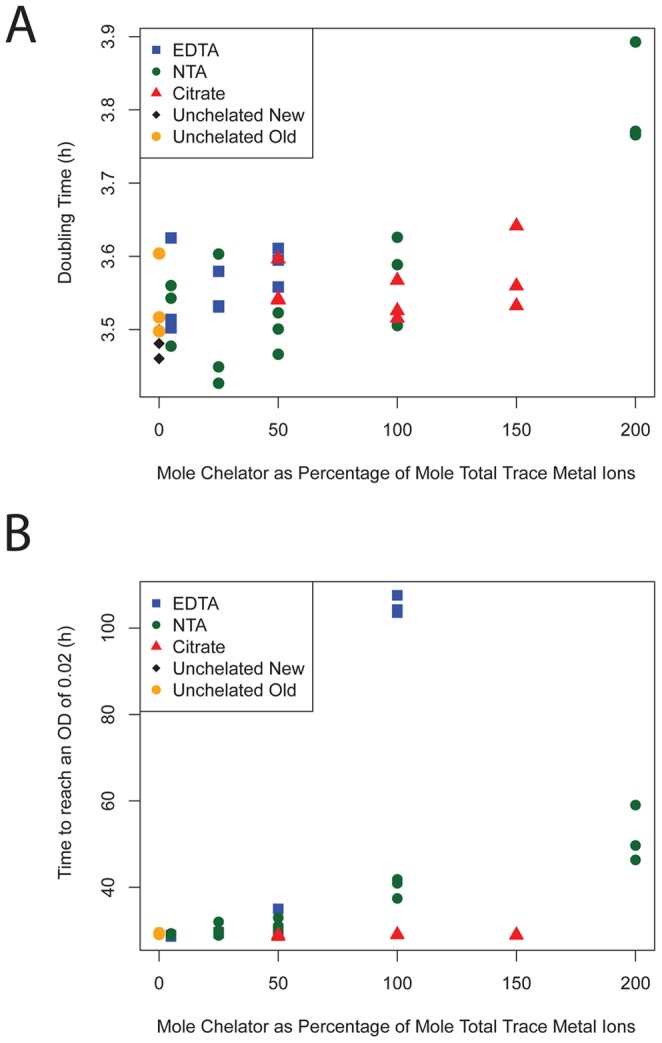
Doubling times of AM1Δ*cel* growing on methylamine using different treatments of chelator and chelator concentration. The concentration of each chelator is shown on the x-axis as a percentage ratio of the moles of the chelator relative to moles of the total trace metal ions (i.e., excluding calcium and magnesium; 100% = 25.5 µM). A) The doubling times for the cells under different conditions. Doubling times are not shown for cells growing at 100% or 200% EDTA as these treatments either never increased in OD (the 200% treatment) or did so after a significantly longer time than the other treatments and never achieved a high enough OD to accurately measure a growth rate (the 100% treatment). B) Time taken for cultures to reach an OD of 0.02 in hours (calculated by interpolation of the growth curve).

We found that neither EDTA nor NTA were acceptable chelator options, but that either citrate or no chelator were. On methylamine, except for the 100% and 200% EDTA treatments, AM1 was able to grow on all chelated and unchelated medium (Fig 2A). Cultures of the 200% EDTA treatment never grew and the 100% EDTA treatments began to only exhibit very slow growth after ∼85 hours (Fig 2B). These two treatments were therefore excluded from further analysis. We analyzed the remaining treatments using a linear model where growth rate was a function of the term for chelator treatment and an interaction between each chelator and the relative concentration of chelator. In agreement with the general pattern in Fig 2A, which shows slower growth at higher NTA concentrations, the only statistically significant term below a 0.05 threshold in this model was the interaction between NTA and chelator concentration (p = 0.01), evincing that NTA reduces growth rate as its concentration increases (Fig 2B).

On succinate, the chelator used had less of an effect upon growth. The only meaningful difference was observed for the 200% EDTA treatment. This treatment was much worse than the other treatments having a mean growth rate 6.7 units of the standard error away from the mean of all other treatments on AM1Δ*cel*, and 3.85 standard errors away from the mean of all other treatments on PA1Δ*cel*. We concluded from this experiment that unchelated or citrate chelated metals would not inhibit growth, and chose citrate chelated metals for our medium as they did not form precipitates as the unchelated ones did.

#### 4. PIPES vs. phosphate as a pH buffer

We tested the effect of varying the type of pH buffer used and its concentration on growth rate for all strains (AM1Δ*cel* and PA1Δ*cel*) on all substrates. We ran a full factorial experiment using two buffers, PIPES or phosphate, at two concentrations, either 30 mM or 48 mM. The four possible treatments were added separately to the following additional medium ingredients: 5 mM ammonium sulfate, C7 metals combined with CaCl_2_ solution and 3.33 mM P-Solution (Table S1), which was added as a phosphate source.

The growth rates obtained from each replicate in this experiment are displayed in Figure S2. We analyzed the data separately for each strain and substrate combination using a full linear model with effects for the Buffer and Buffer:Concentration interactions. For each strain/substrate combination, all four treatment groups were significantly different (p-values<0.01) except for PA1Δ*cel* growing with PIPES on succinate, where the growth rate was not significantly different at either 30 mM or 48 mM (p = 0.82). Although PIPES medium at 30 mM had the highest estimated growth rate, the differences were slight, with an estimated effect of 1%–5% of the growth rate depending on the substrate. At 48 mM concentrations, however, the differences between PIPES and phosphate were far more pronounced, with the phosphate buffer causing substantially slower growth (Figure S2). Based on these results, we selected PIPES as our medium’s pH buffer.

#### 5. Effect of sterilization protocol on C7 Metal Solution

We tested whether the method used to prepare the C7 metal solution would affect the growth rate. After making our standard C7 metal mix, we either autoclaved, filter-sterilized, or left it untreated before adding it to the base medium. We grew AM1Δ*cel* on methylamine (the most metal sensitive growth condition) and found no significant differences among the growth rates measured in these three treatments (F_2,19_ = 0.18, p = 0.84). We concluded that metal preparation did not detectably affect growth rate.

#### 6. Excluding citrate as a possible substrate

We tested whether the citrate added as a chelator to the MP medium could be used by AM1Δ*cel* or PA1Δ*cel* as a carbon source for growth. Cultures of AM1Δ*cel* and PA1Δ*cel* were inoculated at 1∶1000 dilution following overnight growth on succinate into either MP medium or MP medium supplemented with a 10 mM concentration of sodium citrate. Each treatment was replicated in 12 different wells. After 54 hours, OD measurements were made of all cultures.

The cultures with additional citrate did not have significantly higher OD measurements for either AM1Δ*cel* (one-sided t = −2.37, p = 0.98) or PA1Δ*cel* (one-sided t = −1.12, p = 0.86). The mean OD measurement for all cultures was also close to 0 (mean = 0.001) and was thus orders of magnitude lower than what is measured after growth on 7 mM succinate.

#### 7. Optimizing concentrations of medium components

Having selected PIPES as the buffer and the citrate chelated C7 solution as the source of trace metals in the new medium, we next tried to ensure that the new medium would be robust to reasonable variations in the exact concentration of all its component solutions (the MgCl_2_, the phosphate, the C7 metal mix combined with the CaCl_2_, and the (NH_4_)_2_SO_4_). We also wanted to look for any further optimizations that might be possible. To do this, we used a full factorial design with a “central point” [27], which is an experimental design that allows one to not only estimate the effect of individually altering the concentration of any component, but also the effect of simultaneously altering several components. We used as the central point the levels of medium components that preliminary experiments suggested was close to optimal (5 mM (NH_4_)_2_SO_4_, 0.25 mM MgCl_2_, 1X C7 metal mix combined with CaCl_2_, and 3.33 mM phosphate). From here, we raised or lowered each component to a higher or lower value to an extent that far exceeds likely differences between replicated preparations of the medium, and made all combinations of these changes. The higher and lower values for each component were as follows: (NH_4_)_2_SO_4_–3 mM and 7 mM; MgCl_2_–0.125 mM and 0.5 mM; C7 metal mix and CaCl_2_–0.5X and 1.5X; and phosphate –2.66 mM and 4 mM. This resulted in 17 treatments (2^4^ = 16 full factorial variants plus 1 central value). Each set of media components was made independently in 3 different batches and replicated 3 times (n = 153 for each strain/treatment) for both strains on both substrates. In addition, because each plate can hold only 48 samples, the plate/incubator-position was included as a blocking covariate in the analysis (henceforth referred to as the “Slot” factor).

We found that growth was very robust to the large changes in the concentrations of the different components that we tested. The estimated standard error in a linear model fit to the specific treatment and blocking plate factor (i.e. the pure error) was between 1% and 2% of the estimated growth rate at the central point for every strain and substrate tested, the data were consistent across different treatments. Using the same model, the greatest difference in estimated growth rates (defined as the growth rate of the fastest estimated treatment subtracted from the slowest estimated treatment) was only 4%, 6% and 9% for AM1Δ*cel* on methylamine, AM1Δ*cel* on succinate and PA1Δ*cel* on succinate, respectively.

To determine which medium components contributed to the small amount of variation observed across treatments and to discover any further optimizations, we analyzed data from each strain and substrate combination using linear models with the concentration of each media component as a factor coded using a −1, 0, +1 scheme. Full models with complete interactions for all components as well as the slot blocking variable were initially used to fit the data. We ensured that any conclusions held over a variety of models that could be reduced from the complete model using a combination of approaches including the Akaike Information Criterion (AIC, which measures the goodness of fit balancing for model accuracy and complexity) and the effect-hierarchy principle (which searches for interactions with any main-effects found to be significant). We also examined partial residual plots to evaluate any need for quadratic terms in the model.

For all strain and substrate combinations, we found that the concentrations of (NH_4_)_2_SO_4_ and MgCl_2_ were the only two statistically significant factors, though their estimated effect sizes were very small. Increasing the MgCl_2_ relative to the lowest level tested appeared to be mildly beneficial for all strains on all substrates. When AM1Δ*cel* was grown on methylamine the estimated effect size was less than 0.5% of the estimated growth rate at the central point in the experimental design (MgCl_2_ = 0.001, p = 0.0001 in model: GrowthRate ∼ Slot+MgCl_2_+ (NH_4_)_2_SO_4_). The effect was less than 1% of the central point’s growth rate when the strain was grown on succinate (MgCl_2_ = 0.0024, p = 3.13×10^−6^ in model: GrowthRate ∼ Slot+MgCl_2_+ (NH_4_)_2_SO_4_). For PA1Δ*cel* grown on succinate, the main effect of increased MgCl_2_ concentration was only 2% of the central point’s growth rate, though this interpretation is slightly complicated by an interaction term in the model that slightly reduces the total beneficial effect when the ammonium concentration is also increased (MgCl_2_ = .0055, p<2×10^−16^ in model: GrowthRate ∼ Slot+MgCl_2_+ (NH_4_)_2_SO_4_+ MgCl_2_: (NH_4_)_2_SO_4_).

As increased MgCl_2_ resulted in faster growth_,_ we sought to optimize its concentration with an additional experiment that used a range of higher concentrations. We grew all strains and substrates in media with MgCl_2_ concentrations at concentration above the previous lowest value of 0.125 mM at concentrations of 0.25, 0.5, 0.75, 1.5 or 2 mM, with all other media components set to the midpoint of the previously tested range. Although we found no significant effect in this later experiment for the concentration of MgCl_2_ with PA1Δ*cel* (F_1,39_ = 0.84, p = 0.37), with AM1Δ*cel* there appeared to be a very slight negative effect of increasing MgCl_2_ on either substrate (p-values in both regressions <0.05, with both estimated effect sizes less than 2% of the mean growth rate per mM increase in MgCl_2_). This negative effect was largely due to a decrease in the growth rate that began at the 1.5 or 2 mM levels and no significant differences were found when comparing the mean growth rates across the 0.25–0.75 mM range for any strain or substrate concentration (p-values >0.5). We concluded from these experiments that the growth rate slightly increases with MgCl_2_ concentrations up to 0.125 mM, plateaus, and then begins to decrease at concentrations above 1.5 mM. This result is consistent with a previous study that found MgCl_2_ limited the growth of AM1 on methanol at concentrations below 0.121 mM [38]. For this reason, we set the MgCl_2_ level at 0.5 mM in MP medium, a level on the plateau between the beneficial and detrimental effects, and kept the concentration of all other medium components at the midpoint of their tested levels in MP medium.

In contrast to the consistently beneficial effect of increased MgCl_2_, the effect of increasing the (NH_4_)_2_SO_4_ concentration was very slightly deleterious on methylamine and very slightly beneficial on succinate. Effect sizes using the previously specified statistical models were as follows: succinate AM1Δ*cel* = 0.001, PA1Δ*cel* = 0.004; methylamine: AM1Δ*cel* = −0.0009; all 3 p-values for each estimated effect <0.0006). As the effect size in all cases was less than 0.4% of the mean growth rate for each strain and substrate, and because the direction of the effect depended on the substrate, we did not further consider this variable for optimization. One possible explanation for this divergent result is that ammonia is liberated during methylamine consumption, and thus additional nitrogen in MP medium is unnecessary for growth on this substrate.

#### 8. Comparison to other media

To validate that our medium, henceforth “MP”, compared well to other formulations currently used to grow *M. extorquens*, growth rate was compared for AM1Δ*cel* growing on methylamine and methanol, as well as AM1Δ*cel* and PA1Δ*cel* on succinate, in MP and four other media. The first medium we tested was our historically used variant-Hypho (aged for over four weeks). The second and third media tested were phosphate-buffered media that differed in initial pH (second media: initial pH = 6.7 for growth on multi-carbon compounds, henceforth “Phosphate-multi-C”; third media: initial pH = 7.1 for growth on C_1_ compounds, here “Phosphate-C_1_” [39]). The rationale behind testing different pH levels was to partially counter the tendency that growth on multi-C substances increases pH, whereas the opposite is seen for C_1_ compounds. The final medium we compared, Choi medium [40], is a *Methylobacterium* medium developed to aid poly-β-hyroxybutyric acid (PHB) production and has an exceptionally metal-rich formulation; total trace metals are in the mM range instead of the µM range. A comparison of the concentrations of main components of each of these media is given in Table 2.

**Table 2:** Comparison of the main components of the different mediums.

	Old Media (Hypho-Variant)	Phosphate C_1_	Phosphate Multi-C	MP	Choi
**Buffer**	Phosphates	Phosphates	Phosphates	PIPES	Phosphates
**pH**	6.73	7.1	6.7	6.75	6.8
**Buffer Conc.**	33.3 mM	20.7 mM	20.7 mM	30 mM	24.6 mM
**Chelator**	EDTA	EDTA	EDTA	Citrate	None
**Calcium**	9.98 µM	20.41 µM	20.41 µM	20 µM	13.6 mM
**Total Metals** **(excluding Ca and Mg)**	12.66 µM	63.16 µM	63.16 µM	25.53 µM	13 mM
**Nitrogen**	1.89 mM	30.29 mM	30.29 mM	4 mM	5.68 mM
**Phosphates**	33.3 mM	20.7 mM	20.7 mM	3.33 mM	24.6 mM
**Magnesium**	0.81 mM	0.81 mM	0.81 mM	0.5 mM	1.83 mM

**Figure 3:**
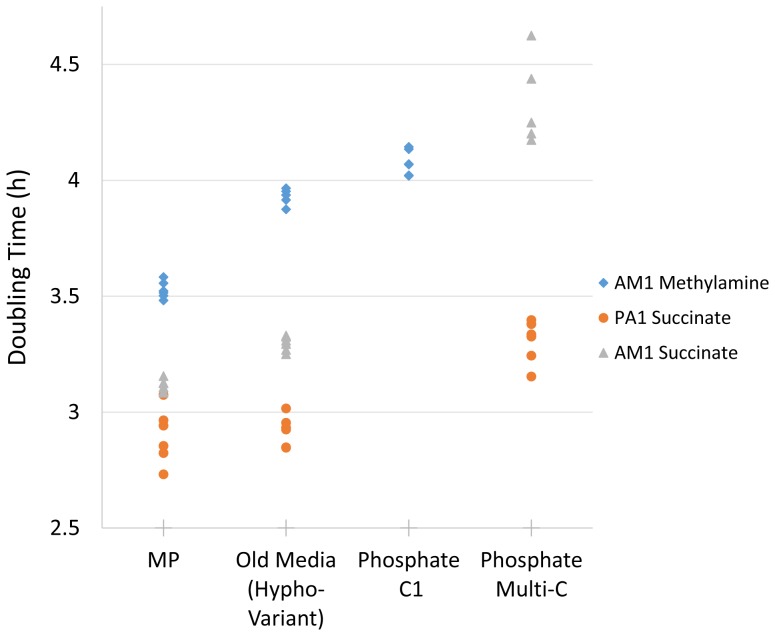
Growth rates of AM1Δ*cel* and PA1Δ*cel* on different media formulations and carbon sources. Measurements were taken every 50 minutes. The data for the Choi medium is not shown because the high concentrations of metals altered the OD readings (Fig. S3) such that meaningful growth rate comparisons could not be made.

On C_1_ compounds, strains grown in MP medium grew faster than in all other media (Fig. 3). With methylamine as the substrate, the growth rate on MP was estimated to be 11% faster than on our older variant-Hypho, and 15% faster than on Phosphate-C_1_ medium (all p-values <1×10^−6^). With methanol as the substrate, due to evaporation, the cultures did not achieve an OD over 0.1 and could not be fit over the same range of OD values; however, when fit over an OD range of 0.01–0.07, the MP medium was estimated to be 7% and 17% faster than on variant-Hypho and Phosphate-C_1_ (p-values <1×10^−6^), respectively. We did not make comparisons to the Choi media as it produced data that was too noisy for meaningful analysis (Fig. S3). Although the Choi medium did appear to have growth rates similar to the other media tested, the large concentration of unchelated metals in Choi medium formed dense precipitates on the bottom of the wells, making it difficult to set a well’s blank value and causing erratic OD measurements throughout the growth period (Fig. S3). For this reason, meaningful quantitative comparisons could not be made and we concluded that Choi medium could not be used for growth rate measurements in microtiter plates (Fig. S3).

On succinate, strains grown in MP medium performed as well as or better than the other media we tested (Fig. 3, Fig. S4 **)**. For AM1Δ*cel* growing on succinate, the mean growth rate was estimated to be 6% faster with MP as compared to Hypho medium (p = 1.74 ×10^−8^), while for PA1Δ*cel* there was a smaller but still significant improvement of 1.7% compared to Hypho medium (p = 0.01). Although the Phosphate-multi-C medium initially appeared to grow similarly to variant-Hypho or MP, its growth rate noticeably slowed as OD increased in a clear violation of the exponential growth model (Fig. S4) and so it was not quantitatively compared to the other media.

## Discussion

We have developed a high throughput system to accurately and reproducibly measure growth rates of *M. extorquens* strains by integrating robotic instruments, genetically modifying strains and designing a new growth medium. Our system is capable of simultaneously measuring the growth rate of 1,920 cultures of *M. extorquens* if each 48-well plate is measured every 50 minutes. The deletion of the *cel* operon in both *M. extorquens* strains was a large step towards being able to make such accurate measurements. Even with otherwise optimal growth conditions, the formation of clumps of grouped cells (Fig 1A) that still contained these genes obscured the relationship between increasing OD measurements and the total increase in biomass, and led to data that was too noisy to allow for precise growth rate measurements.

The use of 48-well plates instead of 96-well plates was another important factor and is a substantial difference between our system and others. It was also the only change that required us to custom fabricate components for our robotic system rather than simply combine available products; the slots that hold plates in the incubator tower had to be redesigned to fit the larger plates. Although a previous study also found that 48-well plates provide significantly better mixing [41], we were very surprised that the two types of nearly identical 48-well plates we tested had such drastically different mixing and growth characteristics. These two plate types have indistinguishable standard dimensions and are both made of polystyrene. However, the CoStar plates are tissue culture treated, while the Greiner plates are not, and we suspect this explains the difference in how the medium swirled in their wells and whether they allowed for stable growth rates.

The new medium formulation optimized for *M. extorquens*, MP, overcomes inconsistencies in other media and is robust to minor variations in its components. There are several aspects of this media that make it robust relative to other media. A major difference between MP and other media commonly used for *M. extorquens* is the decision to use citrate instead of EDTA as a chelator. Although EDTA had clear disadvantages and did not allow for consistent measurements, because a citrate chelator could be a possible carbon source for some organisms it is sometimes avoided. Notably, citrate is only present in MP at a concentration of 45 µM, which is 100-fold or more below substrate concentrations utilized for growth [42]. However, our results indicate that using citrate does not affect growth dynamics; when the total concentration of the C7 solution was varied as part of our experiments no differences were found in the growth rate. Furthermore, no growth was detected after 54 hours with 10 mM citrate, suggesting that this compound does not function as a growth substrate for either AM1 or PA1. We also found citrate preferable to not using any chelator. Although cultures appeared to grow as fast on an unchelated version of the C7 metal mix (made by simply excluding the citrate), the oxidation state of metal cations in a liquid solution can more readily change if they are not chelated, and at equilibrium oxidized and unchelated metals may almost be entirely in biologically unavailable forms if they have largely precipitated out of solution [43]. The unchelated C7 metal mix appears susceptible to these problems as it does form a precipitate, making it difficult to ensure consistent concentrations in different aliquots, and it also changes color as it ages over several months. Thus, in designing a new medium, we have chosen to use citrate as the chelator due to better optical properties and greater stability.

The MP medium, similar to other media used for *Methylobacterium* species [40], [44], [45], is unusually metal rich. Many of the metals in it are present at concentrations above 1 µM, whereas most media for bacteria provides each trace metal at a concentration between 0.01 and 1 µM, as they are often toxic at higher concentrations [30]. The higher metal concentrations in MP medium are not toxic to *M. extorquens* however, as we found no advantage on either succinate or methylamine to increasing or decreasing the concentrations by 50%.

Interestingly, we found that growth on single carbon compounds requires higher metal concentrations than growth on multiple carbon compounds and this likely explains why growth on succinate is relatively unaffected by the type of chelator used. In preliminary studies leading up to the work presented here we found that when AM1 grows on succinate, the concentration of the C7 solution can be reduced to a small fraction of its level in MP medium without affecting the growth rate. In contrast, the metals must be maintained much closer to the unusually high concentrations in MP medium for growth on single carbon compounds. In particular, for growth on methylamine, copper, a component of the amicyanin protein thought to receive electrons from methylamine dehydrogenase [46] was found to be the first metal to limit growth in earlier tests. However, we did not find that all metals were required in measurable concentrations. As an example, the C7 metal solution, unlike the other media we compared, also contains tungsten as a component. We wanted to ensure that tungsten was available in adequate amounts as it has been shown to be used by a formate dehydrogenase enzyme in *M. extorquens* [47]. However, we were unable to show either a positive or negative effect of explicitly adding tungsten to the medium, as equivalent growth rates are obtained with or without it, implying that some ambient source of tungsten is usually sufficient or the cofactor is unnecessary.

Although the final MP medium formulation for both AM1Δ*cel* and PA1Δ*cel* is robust to large deviations in the concentrations of almost all its components, the exception is a trade-off when selecting the concentration of the pH buffer. Low buffer concentrations can create initially faster growth rates, while higher concentrations allow for a slower but more consistent growth rate over a larger range of OD values. In this study we only demonstrated that growth rate decreases with an increasing buffer concentration by comparing growth rates at concentrations of either 30 or 48 mM, but preliminary work suggested that the growth rate of *M. extorquens* appeared to be slightly faster when the concentrations of the pH buffer was below 30 mM. However, it is difficult to take reliable growth rate measurements of cultures grown in concentrations below 30 mM as the growth rate significantly declines as the culture grows, making it hard to measure any single consistent growth rate. This was also seen in the Phosphate-multi-C medium we tested, which uses a 20.7 mM concentration of buffer leading to a decreasing growth rate at higher OD values (Fig. S3)**.** We chose a 30 mM buffer concentration (with an additional 3.33 mM of buffering provided by the phosphate solution) for MP medium as a compromise that allowed stable measurements over a range of OD values the growth curve, but did not appear to significantly hinder growth relative to lower concentrations.

At a 30 mM buffer concentration, using PIPES instead of a phosphate buffer only slightly increases the growth rate. Although the two buffers behaved similarly, we selected the slightly harder to prepare PIPES buffer because the phosphate had a tendency to occasionally form a small amount of white “snow” in the medium (presumably calcium phosphate precipitates). This snow not only changes the composition of the medium but also could lead to false positive counts if cells were being counted by flow-cytometry. Furthermore, at higher buffer concentrations PIPES is clearly superior to phosphate, particularly on succinate (Fig. S3). MP medium could be altered to grow *M. extorquens* at higher densities by increasing the PIPES concentration without seeing as substantial a decrease in the growth rate, unlike if a phosphate buffer was equivalently increased. This may be applicable to current media formulations designed to optimize the production of industrial products using *Methylobacterium* [44], [45], and these media might benefit by switching the buffer from phosphate to PIPES. It is also possible that our strains with the deleted cellulose operon would make better candidates for industrial production strains, as presumably less biomass is being channeled towards production of extra-cellular carbon.

Our medium, strains and instrumentation allows for precise measurements of growth rates at a large scale. As the study of the physiology of *M. extorquens* and other model systems has become increasingly quantitative, the need to move beyond the “-, +/−, +, ++” categorization of microbial growth to more precise measurements of the growth rate has become ever more important. Interesting questions that can now be more effectively answered range from exploring the growth of strains across a wide spectrum of continuously-varied enzyme levels via a regulated promoter [4] to exploring differences in the lag time required to switch between substrates [13]. Furthermore, from an evolutionary perspective small differences in the growth rate can be tremendously important. Beneficial mutations in populations that have evolved in batch culture, where growth rate is the primary selective component, can commonly be less than 10% and as adaptation proceeds the average selective effects tend to decrease. Being able to evolve strains in a consistent media environment and measure their growth rates as accurately as our system allows can therefore provide great insight into the adaptive dynamics of evolving populations.

## Supporting Information

Figure S1 **OD through time for AM1Δ** ***cel*** **grown in a 96-well plate on MP media with succinate.** Readings were taken approximately every 50 minutes.
(PDF)

Figure S2 **Comparison of the growth rates on different pH buffers.** Shown are AM1Δ*cel* and PA1Δ*cel*, grown on one of two substrates (methylamine or succinate) with either PIPES or Phosphate used as a buffer at one of two concentrations. Red symbols indicate 48 mM buffer concentration and black symbols indicate 30 mM buffer concentrations of both PIPES and Phosphate buffered media. Initial pH of all media formulations was 6.7.
(PDF)

Figure S3 **Precipitates and growth readings in Choi medium.** A) The bottom of Falcon tubes containing 6 ml of either Choi medium (not centrifuged, green) or MP medium (red). The high concentration of metals in the Choi medium leads to formation of a large amount of precipitates. B) Two growth curves of AM1Δ*cel* growing on either Choi medium (green) or MP medium (red). The precipitates in Choi cause higher initial OD readings and noisier data.
(PDF)

Figure S4 **OD through time plots of AM1Δ** ***cel*** **growing on succinate (5.6 mM) in three different media.** The growth rate in the Phosphate multi-C medium decreases during growth and the final OD is lower compared to the other two media treatments.
(PDF)

Table S1 **The recipe for the new MP medium and C7 trace metal mix.**
(PDF)

Text S1 **Recipe for variant-Hypho medium.**
(PDF)
