# Supplementary figures and images for: Development of an Optimized Medium, Strain and High-Throughput Culturing Methods for Methylobacterium extorquens
Source: PLoS One. 2013 Apr 30;8(4):e62957. doi: 10.1371/journal.pone.0062957 (PMC3639900; doi:10.1371/journal.pone.0062957)

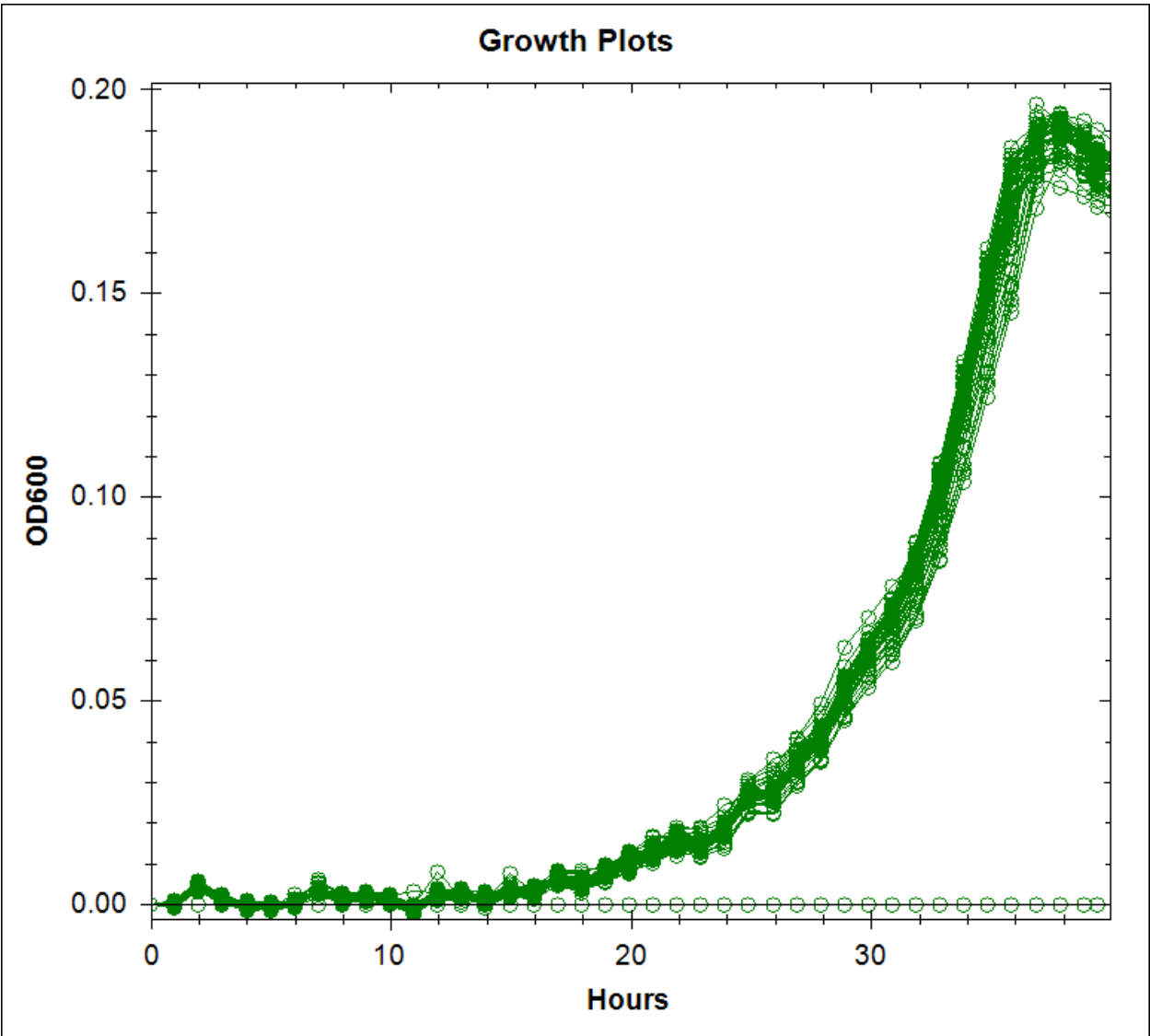

Supplement: Figure S1 — OD through time for AM1Δ cel grown in a 96-well plate on MP media with succinate. Readings were taken approximately every 50 minutes. (PDF) [file pone.0062957.s001.pdf]

PA1 on Succinate

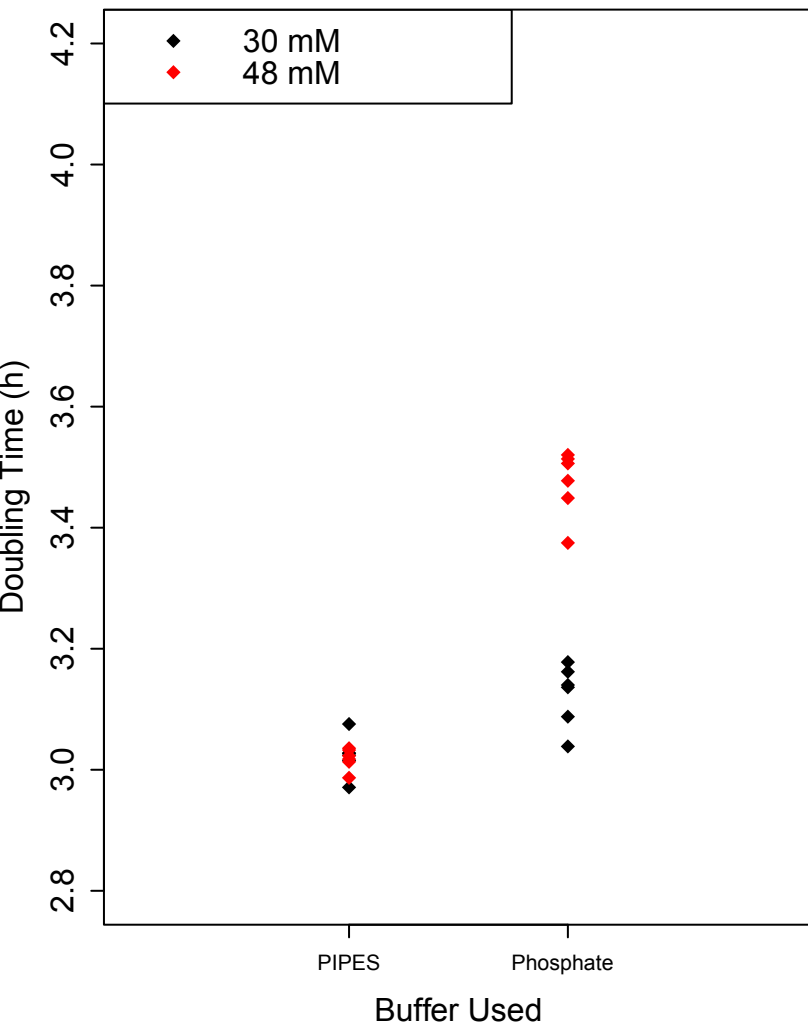

AM1 on Succinate

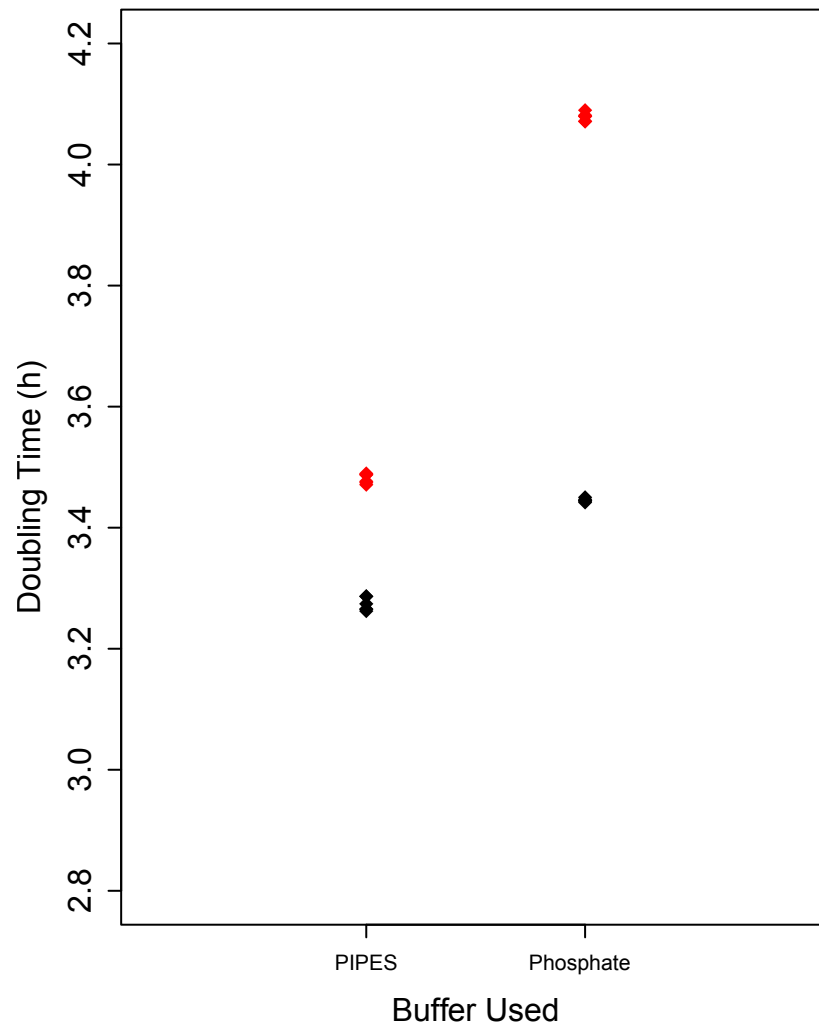

AM1 on Methylamine

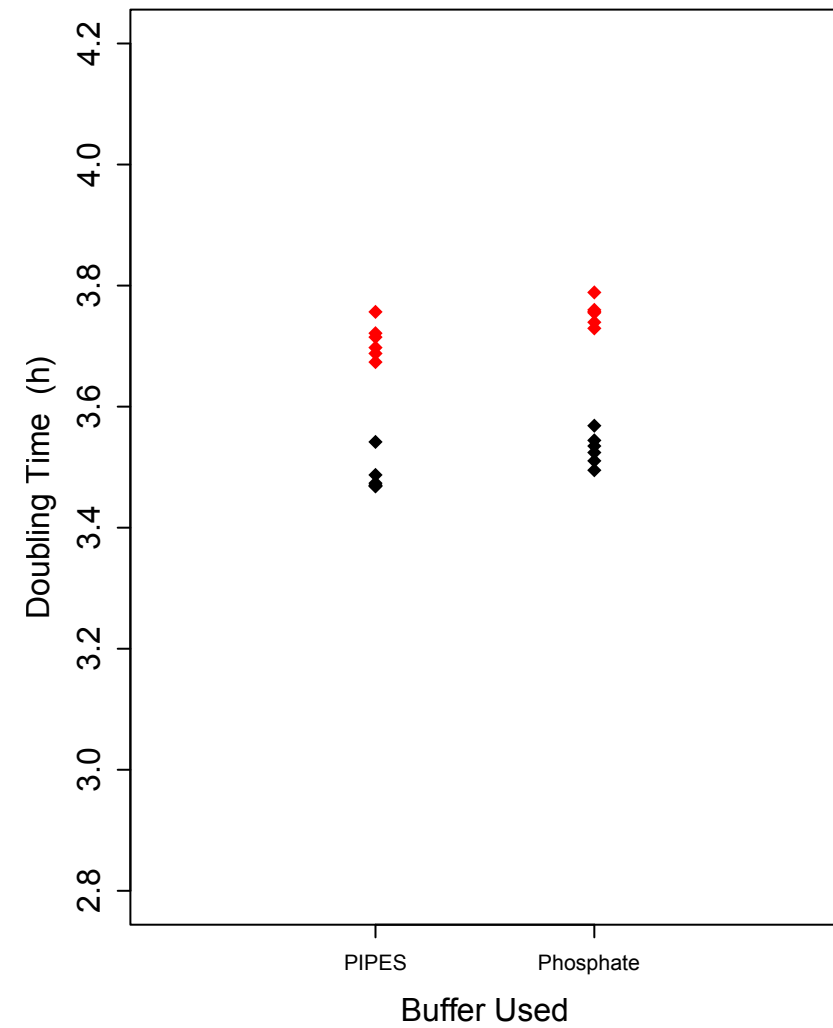

Supplement: Figure S2 — Comparison of the growth rates on different pH buffers. Shown are AM1Δcel and PA1Δcel, grown on one of two substrates (methylamine or succinate) with either PIPES or Phosphate used as a buffer at one of two concentrations. Red symbols indicate 48 mM buffer concentration and black symbols indicate 30 mM buffer concentrations of both PIPES and Phosphate buffered media. Initial pH of all media formulations was 6.7. (PDF) [file pone.0062957.s002.pdf]

A.

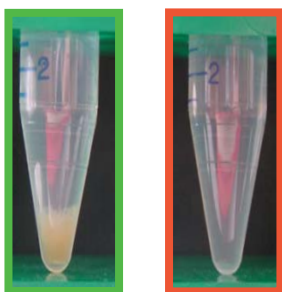

B.

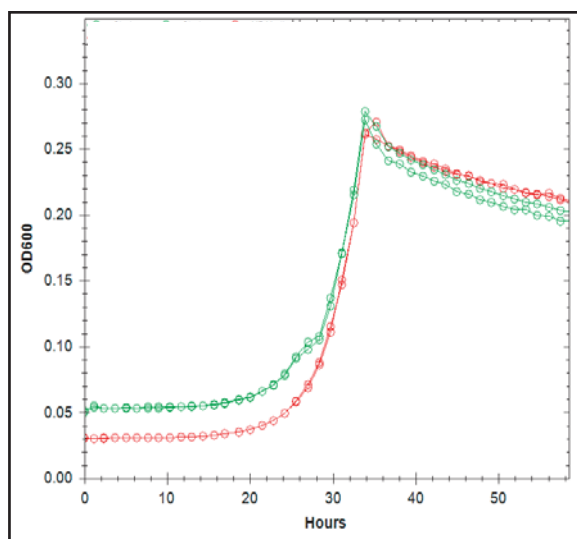

Supplement: Figure S3 — Precipitates and growth readings in Choi medium. A) The bottom of Falcon tubes containing 6 ml of either Choi medium (not centrifuged, green) or MP medium (red). The high concentration of metals in the Choi medium leads to formation of a large amount of precipitates. B) Two growth curves of AM1Δcel growing on either Choi medium (green) or MP medium (red). The precipitates in Choi cause higher initial OD readings and noisier data. (PDF) [file pone.0062957.s003.pdf]

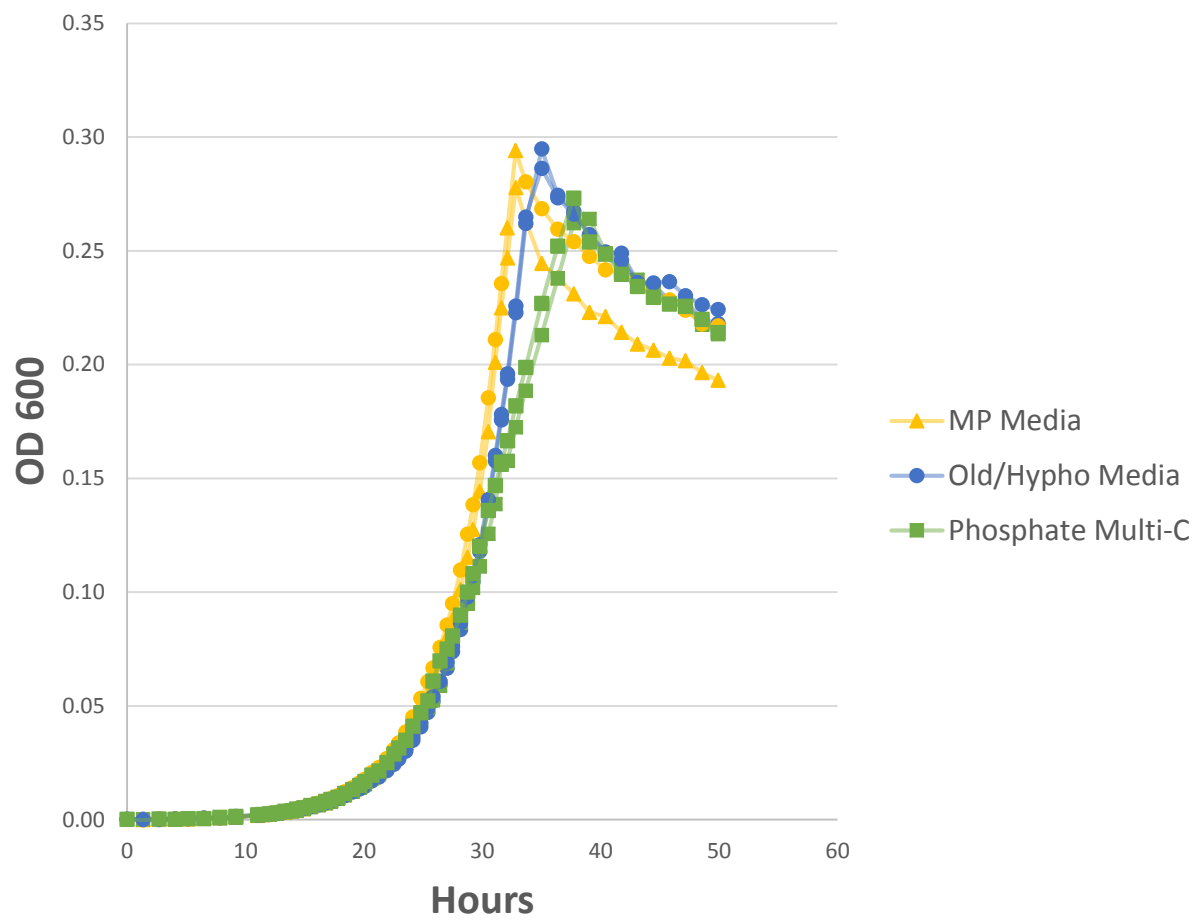

Supplement: Figure S4 — OD through time plots of AM1Δ cel growing on succinate (5.6 mM) in three different media. The growth rate in the Phosphate multi-C medium decreases during growth and the final OD is lower compared to the other two media treatments. (PDF) [file pone.0062957.s004.pdf]
