# Supplementary material for: Development of an Optimized Medium, Strain and High-Throughput Culturing Methods for Methylobacterium extorquens
Source: PLoS One. 2013 Apr 30;8(4):e62957. doi: 10.1371/journal.pone.0062957 (PMC3639900; doi:10.1371/journal.pone.0062957)
Supplement: Table S1 — The recipe for the new MP medium and C7 trace metal mix. (PDF) [file pone.0062957.s005.pdf]

Table S1: Recipe for MP Medium

**MP Media Recipe**			**FOR STOCK SOLUTIONS:**		**FOR 1 L MEDIA:**	
		Molecular Weight (g)	CONCENTRATION	ADD TO 1 L dH_2O	FINAL CONCENTRATION	ADD
**PIPES**	$C_8H_8N_2O_6S_2$	302.37	300 mM (10X)	90.711 g	30 mM	100 mL
**P solution**			(100X)			
	K_2HPO_4 •3 H_2O	228.22	145.0 mM	33.1 g	1.45 mM	10 mL
	NaH_2PO_4 •H_2O	137.99	187.69 mM	25.9 g	1.88 mM	
**$MgCl_2$**	$MgCl_2$ •6 H2O	203.3	2 M (4000X)	406.6 g	0.5 mM	250 µL
**$(NH4)_2SO_4$**	$(NH4)_2SO_4$	132.14	2 M (250X)	264.28 g	8 mM	4 mL
**$CaCl_2$**	$CaCl_2$ •2 H_2O	147.02	2 M (100,000X)	294.04 g	20 µM	10 µL
**C7-Metals**	sodium citrate		(1000X)	ADD TO 500 mL dH_2O		
MIX IN ORDER LISTED ↓	($Na_3C_6H_5O_7$ •2 H_2O)	294.1	45.53 mM	6705.5 mg	45.6 µM	
	$ZnSO_4$ •7 H_2O	287.54	1.2 mM	172.52 mg	1.2 µM	
	$MnCl_2$ •4 H_2O	197.9	1.0 mM	99 mg	1 µM	
	$FeSO_4$ •7 H_2O	278.01	18 mM	2502 mg	18 µM	1 mL
	$(NH_4)_6Mo_7O_{24}$ •4 H_2O	1235.86	2 mM	1235.6 mg	2 µM	
	$CuSO_4$ •5 H_2O	249.68	1 mM	124.8 mg	1 µM	
	$CoCl_2$ •6 H_2O	237.93	2 mM	237.9 mg	2 µM	
	Na_2WO_4 •2 H_2O	329.85	0.33 mM	54.4 mg	0.33 µM	
**milliQ-H_2O**						885 mL

Directions to prepare medium:

1. Make PIPES according to the preceding table and adjust to pH 6.75 by adding KOH.
2. Make additional stock solutions for P, $MgCl_2$, $(NH_4)_2SO_4$. Autoclave all.
3. To make stock solution for C7 METALS, mix metals in order listed, dissolving sodium citrate first. Dissolve each metal before adding the subsequent one. Autoclave.
4. To make media, mix all components together except $CaCl_2$. Autoclave. Add $CaCl_2$. ($CaCl_2$ is added afterwards to avoid calcium phosphates from being formed in the autoclave).
