# Supplementary material for: Development of an Optimized Medium, Strain and High-Throughput Culturing Methods for Methylobacterium extorquens
Source: PLoS One. 2013 Apr 30;8(4):e62957. doi: 10.1371/journal.pone.0062957 (PMC3639900; doi:10.1371/journal.pone.0062957)
Supplement: Text S1 — Recipe for variant-Hypho medium. (PDF) [file pone.0062957.s006.pdf]

Text S1: Recipe for variant-Hypho medium

Concentrations in Final Medium

Chemical	Concentration	Purpose
K_2HPO_4	14.5 mM	Buffer/Nutrient
NaH_2PO_4	18.8 mM	Buffer/Nutrient
$(NH_4)_2SO_4$	3.8 mM	Nutrient
$MgSO_4$	0.8 mM	Nutrient

General Comments

This medium uses phosphates as the buffer and the pH of the final media is determined by the relative concentration of the monobasic and dibasic phosphate components. A media equivalent to this one can also be made by switching the cation used in the phosphates as long as the relative concentration stays the same (such that $K_2HPO_4 \rightarrow Na_2HPO_4$ and $NaH_2PO_4 \rightarrow KH_2PO_4$). This simple recipe does not include calcium or trace metals. Historically, 1000X of a modified Vishniac trace metal mix has been added, as shown below.

Preparation

This media can be prepared by combining two stock solutions.

Recipe (for 1 L):
100 mL 10X P-solution
100 mL 10X S-solution
800 mL deionized H_2O

P-solution (10x):
K_2HPO_4 25.3 g (or 33.1 g $K_2HPO_4 \bullet 3\ H_2O$)
NaH_2PO_4 22.5 g (or 25.9 g $NaH_2PO_4 \bullet H_2O$)
in 1 L of deionized H_2O

S-solution (10x):
$(NH_4)_2SO_4$ 5 g
$MgSO_4 \bullet 7\ H_2O$ 2 g (or 0.98 g $MgSO_4$)
in 1 L of deionized H_2O

Vishniac Trace Elements (1000X):

Add in the following order, adjusting the pH to 5.0 with each addition.

dH_2O	500 mL
EDTA	5 g (or 6.37 g of EDTA •2 H_2O)
$ZnSO_4$ •7 H_2O	2.2 g
$CaCl_2$ •2 H_2O	0.733 g
$MnCl_2$ •$4H_2O$	0.506 g
$FeSO_4$ •7 H_2O	0.499 g
$(NH_4)MO_7O_{24}$ •4 H_2O	0.110 g
$CuSO_4$ •5 H_2O	0.157 g
$CoCl_2$ •6 H_2O	0.161 g

Note: This recipe is based on a trace metal formula given in the fourth footnote of a review paper by Vishniac and Saunter (Vishniac W & Santer M (1957) The Thiobacilli. *Bacteriological Reviews* 21:195.). However, the original formula had 50 fold higher concentrations and used a pH of 6.0.
